# The lncRNA TDRG1 promotes cell proliferation, migration and invasion by targeting miR-326 to regulate MAPK1 expression in cervical cancer

**DOI:** 10.1186/s12935-019-0872-4

**Published:** 2019-05-31

**Authors:** Hui Jiang, Min liang, Yanqiong Jiang, Ting Zhang, Kexin Mo, Suwen Su, Aiping Wang, Yongyi Zhu, Guanqun Huang, Rujian Zhou

**Affiliations:** 1 Department of Abdominal Oncology, The Fifth Affiliated Hospital of Guangzhou Medical University, Guangzhou, 510700 Guangdong China; 2 Department of Gynaecology, The Fifth Affiliated Hospital of Guangzhou Medical University, Guangzhou, 510700 Guangdong China; 3 Department of Clinical Laboratory, The Fifth Affiliated Hospital of Guangzhou Medical University, Guangzhou, 510700 Guangdong China; 4 Department of Anesthesia, The Fifth Affiliated Hospital of Guangzhou Medical University, No. 621 Gangwan Road, Guangzhou, 510700 Guangdong China; 5 Department of General Surgery, The Fifth Affiliated Hospital of Guangzhou Medical University, No.621 Gangwan Road, Guangzhou, 510700 Guangdong China

**Keywords:** lncRNA TDRG1, miR-326, MAPK1, Cervical cancer

## Abstract

**Background:**

Recently, lncRNA-Testis developmental related gene 1 (TDRG1) was proved to be a key modulator in reproductive organ-related cancers. The biological role of TDRG1 in cervical cancer (CC) progression remains largely unknown.

**Method:**

Real-time PCR (qRT-PCR) examined the expression level of TDRG1, microRNA (miR)-326 and MAPK1 mRNA. OS tissues and corresponding relative normal tissues, as well as CC cell lines and normal cell line Ect1/E6E7 were collected to determine the expression of TDRG1 in CC. MTT, colony formation, wound-healing, transwell and flow cytometer assay detected the influence of TDRG1 and miR-326 on CC cells growth, metastasis and apoptosis. Western blot examined proteins level. Bioinformatics, RNA pull-down assay, RNA immunoprecipitation and dual-luciferase reporter assays detected the molecular mechanism of TDRG1 in CC. Xenograft tumour model was established to determine the role of TDRG1 in vivo.

**Results:**

The expression of TDRG1 was significantly increased in CC tissues and cell lines compared with normal tissue and normal cell line respectively and its expression was associated with clinicopathological characteristics of CC patients. Knockdown of TDRG1 inhibited the cell proliferation, migration and invasion in Hela and SIHA cells. Moreover, TDRG1 directly interacted with miR-326, and the inhibition effect on cell growth and metastasis induced by TDRG1 siRNA can be abrogated by miR-326 silencing by its inhibitor in Hela and SIHA cells. Further, MAPK1 was proved to be a direct target of miR-326, and its expression was negatively regulated by miR-326 while positively modulated by TDRG1.

**Conclusion:**

TDRG1 acts as a competing endogenous lncRNA (ceRNA) to modulate MAPK1 by sponging miR-326 in CC, shedding new light on TDRG1-directed diagnostics and therapeutics in CC.

## Background

Cervical cancer (CC) is the second leading cause of cancer-related mortality in female worldwide [1, 2]. According to relevant statistics, nearly 529,800 new cases are diagnosed worldwide annually [3], and the number of CC related death in developing countries are dramatically higher than developed countries [4]. Despite advanced progresses in therapeutic approaches including surgery, radiation and chemotherapy, individually or in combination, a considerable patients’ long-term survival rate still remains suboptimal due to recurrence and metastasis [5]. Hence, it is necessary and urgent to explore novel therapeutic targets or prognosis biomarkers to improve the survival rate of CC patients.

Long noncoding RNAs (lncRNAs) are a family of transcripts with more than 200 nucleotides that lack protein-coding ability and extensively associated with a variety of cancer-related biological activity [6, 7]. Recently, testis developmental related gene 1 (TDRG1) was identified as a novel human testis-specific gene [8], which is a 1.1 kb transcript and locates at 6p212.1-p21.2 spanning 1.18 kb with 2 exons and 1 intron [8]. TDRG1 was initially considered as a key regulator in sperm motility [9], and was involved in the development and progression of testicular germ cell tumors [10]. Further, as a lncRNA, TDRG1 plays important role in cell proliferation, migration and invasion in epithelial ovarian carcinoma [11] and endometrial carcinoma [12]. Based on these findings, TDRG1 has been considered as a critical modulator in several reproductive organ-related cancers, however, the role and potential mechanism of TDRG1 in the tumorigenesis and progression of CC has not been fully investigated.

A growing number of evidences reveal that lncRNAs can act as competing endogenous RNAs (ceRNAs) to regulate cancer-associated gene expression via competitive sharing miRNAs [13, 14]. miRNAs are a class of short non-coding RNAs with approximately ~ 21 nucleotides, and negatively regulate target gene expression by targeting its 3′ untranslated region (UTR) of mRNA [15]. miRNAs are considered as crucial oncogenes or tumor suppressors in regulation of cancer cell growth and differentiation [16]. The interactions between lncRNA and miRNA play key roles in modulation cell growth and metastasis in various cancers [17, 18]. Nevertheless, whether TDRG1 is able to interact with miRNA in CC remains to be explored.

In the present study, we found TDRG1 was directly interact with miR-326 in CC. The expression of miR-326 was decreased and it acted as a tumor suppressor by interaction with lncRNA-HOTAIR or its downstream factors in CC [19–21]. Bioinformatics screening indicated MAPK1 served as a target of miR-326. MAPK1, mitogen-activated protein kinase 1, belongs to MAP kinase family and is a key member of MAPK/ERK pathway [22, 23]. It widely participates in various pathological processes of cancer cells, including cell proliferation, migration, cell differentiation, cell survival and apoptosis [24]. MAPK1 was abundantly expressed and promoted tumorigenicity in CC tissues and cells [25]. Based on the above findings, our study elucidated a novel lncRNA TDRG1/miR-326/MAPK1 regulatory network that provides a potential biomarker and therapeutic target for CC.in the development and progression of CC.

## Materials and methods

### Patients and tissue samples

From March 2016 to August 2017, 30 cervical cancer and 30 normal cervical tissue samples were obtained from The Fifth Affiliated Hospital of Guangzhou Medical University. Tissue samples were collected and immediately snap-frozen in liquid nitrogen and stored at − 80 °C for further analysis. No patients had received chemotherapy or radiotherapy before the surgery. All patients gave their informed written consents prior to the use of these clinical materials for research purpose. The study was approved by The Fifth Affiliated Hospital of Guangzhou Medical University Research Ethical Committee (No. KY2019020128). Written informed consent had been obtained from all participants.

### Cell lines and culture

Human CC cell lines (Hela, SIHA, CaSki, C33A and SW756), and one normal cell line Ect1/E6E7 (a non-cancerous ectocervical epithelial cell line) and the human embryonic kidney cell lines 293 (HEK293) were purchased from Cell Bank of Type Culture Collection of the Chinese Academy of Sciences (Shanghai, China). Cervical cell lines were maintained in RPMI-1640 medium (Gibco, Grant Island, NY, USA), and HEK293 cell line was cultured in DMEM (Gibco). All the mediums were supplemented with 10% fetal bovine serum (FBS, Gibco) and 1% penicillin/streptomycin (Invitrogen, Carlsbad, CA, USA). Cells were cultured in a humidified atmosphere containing 5% CO_2_ at 37 °C.

### Cell transfection

Small interfering RNAs (siRNAs) targeting TDRG1 (siTDRG1#1, siTDRG1#2, siTDRG1#3) and negative controls (siNC), miR-326 mimics for overexpression the miR-326 level and mimics control (NC mimics), miR-326 inhibitor for knockdown the miR-326 level and inhibitor control (NC inhibitor), TDRG1 overexpression plasmid pcDNA3.1-TDRG1 (TDRG1) or its negative control plasmid (Control), as well as TDRG1 knockdown plasmid (shRNA TDRG1) or its negative control (shNC) were purchased from GenePharma (Shanghai, China). Cells transfection were performed using transfection reagent Lipofectamine 2000 (Invitrogen) according to the manufacturer’s instructions. The cells were harvested 24 h after transfection. The target sequences of the siTDRG1#1, siTDRG1#2 and siTDRG1#3 were listed in Table 1.

**Table 1:** List of primer sequences were used in the study

Name	Primer	Sequence (5′–3′)
siTDRG1#1	Sense	5′-CCUUCCCAGGUCUAGGUUCdTdT-3′
	Anti-sense	5′-GAACCUAGACCUGGGAAGGdTdT-3′
siTDRG1#2	Sense	5′-GCGCAGGATCAAGCTACAAdTdT-3′
	Anti-sense	5′-TTGTAGCTTGATCCTGCGCdTdT-3′
siTDRG1#3	Sense	5′-GCTGAGGTTGATCTATTGTdTdT-3′
	Anti-sense	5′-ACAATAGATCAACCTCAGCdTdT-3′
TDRG1	Forward	5′-TCTTCCCTGGCTTGGC-3′
	Reverse	5′-TGGGCTCTTTCGTGGC-3′
GAPDH	Forward	5′-CTCTGCTCCTCCTGTTCGAC-3′
	Reverse	5′-GCGCCCAATACGACCAAATC-3′
MAPK1	Forward	5′-AGGCTGTTCCCAAATGCT-3′
	Reverse	5′-CGTCACTCGGGTCGTAAT-3′
U6	Forward	5′-CTCGCTTCGGCAGCACA-3′
	Reverse	5′-AACGCTTCACGAATTTGCG-3′
miR-326	RT primer	5′-CTCAACTGGTGTCGTGGAGTCGGCAATTCAGTTGAGCTGGAGG-3′
	Forward	5′-ACACTCCAGCTGGGCCTCTGGGCCCTTC-3′
	Reverse	5′-CCAGTGCAGGGTCCGAGGT-3′

### Dual-luciferase reporter assay

The fragments from TDRG1 containing the predicted miR-326 binding site or the corresponding mutants produced by mutating the miR-326 seed region binding site, as well as the fragments from MAPK1 3′UTR containing the predicted miR-326 binding site or the corresponding mutants created by mutating the miR-326 seed region binding site were synthesized by The Beijing Genomics Institute BGI (Beijing, China) and then subcloned into the pmiRGLO Vector (Promega, Madison, WI, USA). HEK293 cells were transfected with TDRG1 Wt or TDRG1 Mut, as well as MAPK1 3′UTR Wt or MAPK1 3′UTR Mut, followed transfected with miR-326 mimics or NC mimics using Lipofectamine 2000 (Invitrogen). The luciferase assay was performed using a dual-luciferase reporter assay system (Promega) according to the manufacturer’s protocol 48 h after transfection.

### Quantitative real time-PCR (qRT-PCR)

Total RNAs were extracted from clinical tissues and using TRIzol reagent (Invitrogen) according to the manufacturer’s protocol. First-strand cDNA were reverse transcribed from RNAs using reverse transcriptase kit (Takara, Otsu, Japan). qRT-PCR was performed using SYBR Green PCR Master Mix (Takara) on a Bio-Rad real-time PCR instrument (Bio-Rad, Hercules, USA). The level of miR-326 was normalized to that of U6. The mRNA expression of TDRG1 and MAPK1 were standardized to control values of glyceraldehyde-3-phosphate dehydrogenase (GAPDH). The relative expression of target genes was calculated with the 2^−ΔΔCt^ method and every sample was prepared in triplicate. The sequence of primers for TDRG1, GAPDH, miR-326, U6 and MAPK1 were listed in Table 1.

### MTT assay

For MTT assay, cells were harvested and seeded in 96-well plates at a density of 1 × 10^3^ cells/ml per well and grew to 80% confluence. Then, 15 μl of MTT solution (5 mg/ml, Sigma, St. Louis, USA) was added into each well and incubated for 4 h at 37 °C. Next, 150 μl DMSO was added into each well to dissolve the formazan crystal. The optical density was measured at 490 nm by spectrophotometry using micro-plate reader (Bio-Tek, Winooski, USA).

### Colony formation assay

For the colony formation assay, cells were plated into 6-well plates. After culture for 14 days, the colonies were fixed with 10% formaldehyde for 30 min and then stained with 0.5% crystal violet for 5 min. The colonies were photographed by a camera (Olympus, Tokyo, Japan).

### Flow cytometer assay

For apoptotic assay, quantification of apoptotic cells was performed using an Annexin-V-FITC apoptosis detection kit (BD, Franklin Lakes, USA). Briefly, transfected cells were digested using trypsin without EDTA, followed resuspended in 500 μl flow cytometry binding buffer. Then, cells were stained with 5 μl Annexin V/FITC and 5 μl propidium iodide (PI) at the room temperature in dark for 15 min. The apoptotic cells were detected by FACS Calibur flow cytometer (BD Biosciences, CA, USA) with the excitation wavelength of Ex = 488 nm and emission wavelength of Em = 530 nm. All experiments were run in triplicate.

For cell cycle analysis, transfected cells (1 × 10^6^) were digested with trypsin and fixed with 70% ice-cold ethanol overnight at − 20 °C. Next, cells were stained with PI (50 μg/ml, Sigma) and RNAse A (0.1 mg/ml, Sigma) for 30 min at 37 °C, and then analyzed by FACS. Each experiment was repeated three times in triplicate.

### Wound-healing assay

Cells were seeded into 6-well culture plates and grew until around 80% confluence. The cell monolayer was scratched with a 10 μl Eppendorf tip. Scratch wounds were recorded using an Olympus microscope (10 × 10) in the same position at 0 h, 24 h. The denuded areas were quantified using Image J software. The wound closure was calculated as followed: (Original width–width of actual wound at 24 h)/Original width × 100%. The experiment was repeated 3 times.

### Transwell assay

Transwell assay was performed for detection of cell migration and invasion. Invasion assay was carried out using transwell chambers (Corning, New York, USA). Cells seeded into the martrigel (Corning, 1 mg/ml)-coated upper chamber were cultured at about 80% confluence and cultured with FBS free medium. The medium with 10% FBS (600 μl) was added to the lower chamber. After 24 h culture, the invaded and migrated cells were fixed with 4% paraformaldehyde and stained with 0.5% crystal violet. Evaluation of invasive and migrative capacity was performed by counting invading cells under an Olympus microscope (40 × 10), and five randomly microscopic views were selected for analysis.

### Western blotting

Total proteins were extracted using RIPA lysis buffer (Sigma) and qualified by a BCA detecting kit (Beyotime Biotechnology, Nanjing, China) according to the manufacturer’s instructions. Protein samples were separated by 10% SDS-PAGE and then transferred onto PVDF membrane (EMD Millipore, Billerica, USA). After incubated in the 5% dry milk blocking buffer for 2 h at room temperature, membranes were incubated with anti-MAPK1 antibody (Abcam, Cambridge, MA, UK; dilution rates of 1:1000), anti-Cyclin D1 antibody (Abcam, dilution rates of 1:100), anti-CDK4 antibody (Abcam, dilution rates of 1:2000), anti-CDK6 antibody (Abcam, dilution rates of 1:1000), anti-Cyclin E1 antibody (Abcam, dilution rates of 1:1000), anti-p-Rb antibody (Abcam, dilution rates of 1:500), anti-Rb antibody (Abcam, dilution rates of 1:2000), anti-Bcl-2 antibody (Abcam, dilution rates of 1:1000), anti-BAX antibody (Abcam, dilution rates of 1:2000), anti-cleaved Caspase 3 antibody (Abcam, dilution rates of 1:500), anti-cleaved Caspase 9 antibody (Abcam, dilution rates of 1:1000), anti-cleaved-PARP antibody (Abcam, dilution rates of 1:1000), anti-PARP antibody (Abcam, dilution rates of 1:1000), anti-E-cadherin antibody (Abcam, dilution rates of 1:2000), anti-N-cadherin antibody (Abcam, dilution rates of 1:1000), anti-MMP-2 antibody (Abcam, dilution rates of 1:1000), anti-MMP-9 antibody (Abcam, dilution rates of 1:1000) and anti-GAPDH antibodies (Abcam, dilution rates of 1:2000) at 4 °C for overnight, respectively. The following day, the membranes were incubated with secondary anti-Rabbit antibody (Abcam, dilution rates of 1:2000) or anti-Mouse antibody (Abcam, dilution rates of 1:2000) at room temperature for 40 min. The blots were measured using an enhanced chemiluminescence detection system (Pierce, Rockford, USA) and signals were captured and intensities of bands were quantified using Image Lab™ Software (Bio-Rad).

### RNA immunoprecipitation assay (RIP)

The Magna RNA-binding protein immunoprecipitation kit (Millipore, Billerica, MA, USA) was used to perform RIP experiments according to the instructions. The RIP assay was performed to explore the binding relationship between endogenous TDRG1 and miR-326 in HEK293 cells. The whole cell lysate was treated with RIP buffer containing magnetic beads conjugated with human anti-Ago2 antibody (Millipore, Billerica, USA), or negative control IgG (Millipore). The samples were incubated with proteinase K with shaking to digest proteins and then the precipitation of RNA was isolated. Purified RNA was extracted and analyzed by qRT-PCR for further study.

### RNA pull-down assay

For RNA pull-down assay, miR-326, miR-326-mutant (miR-326 Mut) with disrupt base pairing between TDRG1 and miR-326 or its negative control (NC) were purchased from GenePharma (Shanghai, China). MiRNAs were biotin-labeled using Biotin RNA Labeling Mix (Roche, Basel, Switzerland) and T7/SP6 RNA polymerase (Roche). Whole cell lysates were mixed and incubated with biotinylated RNAs. Next, the complexes were incubated with Streptavidin agarose beads (Invitrogen) for 1 h at 37 °C. Beads were washed and RNA was analyzed by qRT-PCR.

### Tumor xenograft experiments

Male BALB/c nude mice (4–5 weeks old) were provided by the Model Animal Research Center of Nanjing University (Nanjing, China) and kept under sterile specific pathogen-free (SPF) facility. The protocol was performed according to the Use Committee for Animal Care and approved by The Fifth Affiliated Hospital of Guangzhou Medical University Ethical Committee. To determine the tumorigenic effects in vivo, Hela cells (2 × 10^6^) with stably transfected shRNA TDRG1 or shNC were subcutaneously inoculated into the right flank of the nude mice. Tumor size was measured every 7 days and tumor volume was calculated using the formula V = length × width^2^ × 0.5. After 5 weeks, mice were sacrificed for the further analysis and tumor weight was measured.

### Immunohistochemistry assay (IHC)

For IHC analysis, the xenograft tumor tissues were collected and fixed in 10% formaldehyde, embedded in paraffin and then sectioned in 4 μm thick. The sections were incubated with primary anti-MAPK1 antibody (Abcam, dilution rates of 1:500) and anti-Ki-67 antibody (Abcam, 1:1000) at 4 °C for overnight. Next day, the secondary HRP-conjugated anti-Rabbit antibody was incubated for 1 h at room temperature, and then developed using DAB plus kit. The stained images were captured using an Olympus microscopy.

### Statistical analysis

The data were presented as Mean ± SD of three independent experiments and processed using the Statistical Package for Social Sciences version 17.0 (SPSS 17.0; SPSS, Inc., Chicago, IL) and the Prism statistical software package (Version 5.0; GraphPad Software, Inc.). Student’s *t* test were used to compare differences between the two groups, and multiple group comparisons were analyzed with one-way analysis of variance (ANOVA). Pearson correlation coefficient was used for statistical correlation. Survival curves were evaluated by Kaplan–Meier analysis. A value of *P* < 0.05 was considered statistically significant. All experiments were performed at least three times.

## Result

### TDRG1 was highly expressed in human CC tissues and cell lines

**Fig. 1:**
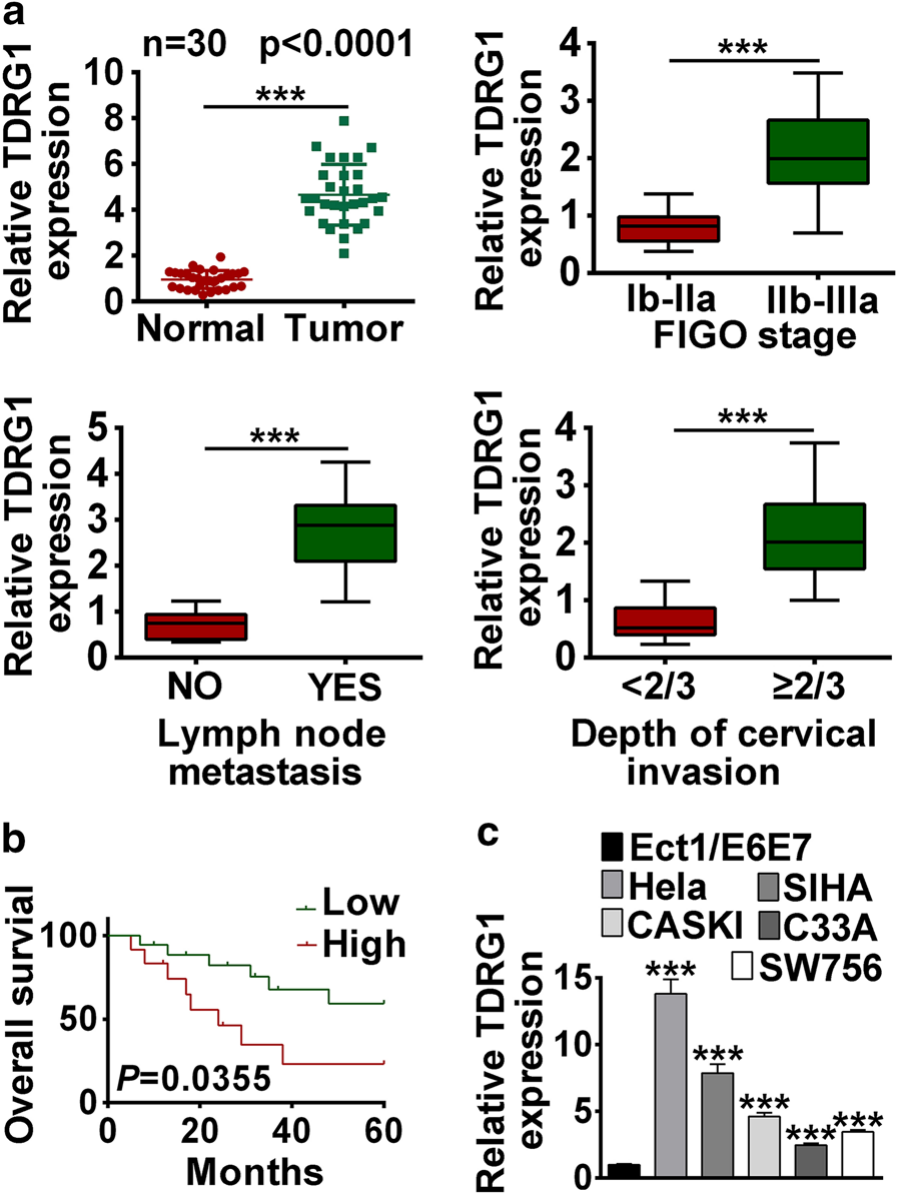
The highly expressed TDRG1 was associated with poor clinical outcome of CC patients. **a** The TDRG1 expression levels in CC tissues and corresponding normal tissues (n = 30) were detected by qRT-PCR. n = 30. The correlation between TDRG1 expression and FIGO stage, lymph node metastasis and depth of cervical invasion were analyzed by qRT-PCR. **b** Kaplan–Meier analysis exhibited the 5-year survival rate of CC patients with high or low expression levels of TDRG1. **c** The TDRG1 expression level in CC cell lines (Hela, CASKI, C33A, SW756 and SIHA) and parallel normal cell line (Ect1/E6E7) were analyzed by qRT-PCR. Data were expressed as mean ± SD. *P < 0.05, ***P < 0.001

To verify the expression levels of TDRG1 in human CC tissues, RNAs were extracted from 30 cases of CC samples and 30 cases of normal paired cervical tissues, and then the expression of TDRG1 was determined by qRT-PCR. The results showed that TDRG1 expressions were increased in cervical tumor tissues compared with normal tissues (*P* < 0.001, Fig. 1a). In addition, the correlation between TDRG1 expression and clinicopathological characteristics (including FIGO stage, lymph node metastasis and depth of cervical invasion) of CC patients were analyzed. The detailed clinicopathologic characteristics of CC patients was shown in Table 2. The elevated expressed TDRG1 was positively correlated with advanced stage (IIb-IIIa), lymph node metastasis (Yes) and depth of cervical invasion (≥ 2/3) in patients (*P* < 0.001, Fig. 1a). Moreover, Kaplan–Meier analysis showed that the strengthened expression of TDRG1 was negatively related with overall survival in CC patients (*P* < 0.05, Fig. 1b). Moreover, the expression levels of TDRG1 were also up-regulated in CC cell lines (Hela, CASKI, SIHA, C33A and SW756) compared with normal cell line (Ect1/E6E7, *P* < 0.001, Fig. 1b). The Hela and SIHA cell lines were selected for the further experiments as the expressions of TDRG1 were higher in Hela and SIHA than CaSki cell lines (Fig. 1b). These data showed that the expression of TDRG1 was upregulated in CC tissue and cell lines, indicating high carcinogenicity in CC patients.

**Table 2:** Correlation between TDRG1 expression level and clinicopathological parameters of CC patients

Clinical parameters	Cases	TDRG1 expression level		x^2^	P
		Low (n = 18)	High (n = 12)		
Age (years)					
≤ 40	8	6	2	–	0.419*
> 40	22	12	10		
FIGO					
Ib-IIa	18	14	4	4.219	0.040
Ib-IIIa	12	4	8		
Tumor size (cm)				0.000	1.000
≤ 4	21	13	8		
> 4	9	5	4		
Differentiation					
Well/moderate	19	15	4	5.748	0.017
Poor	11	3	8		

* Representing Fisher’s precise probability method

### Knockdown of TDRG1 expression inhibited cell proliferation, migration and invasion

**Fig. 2:**
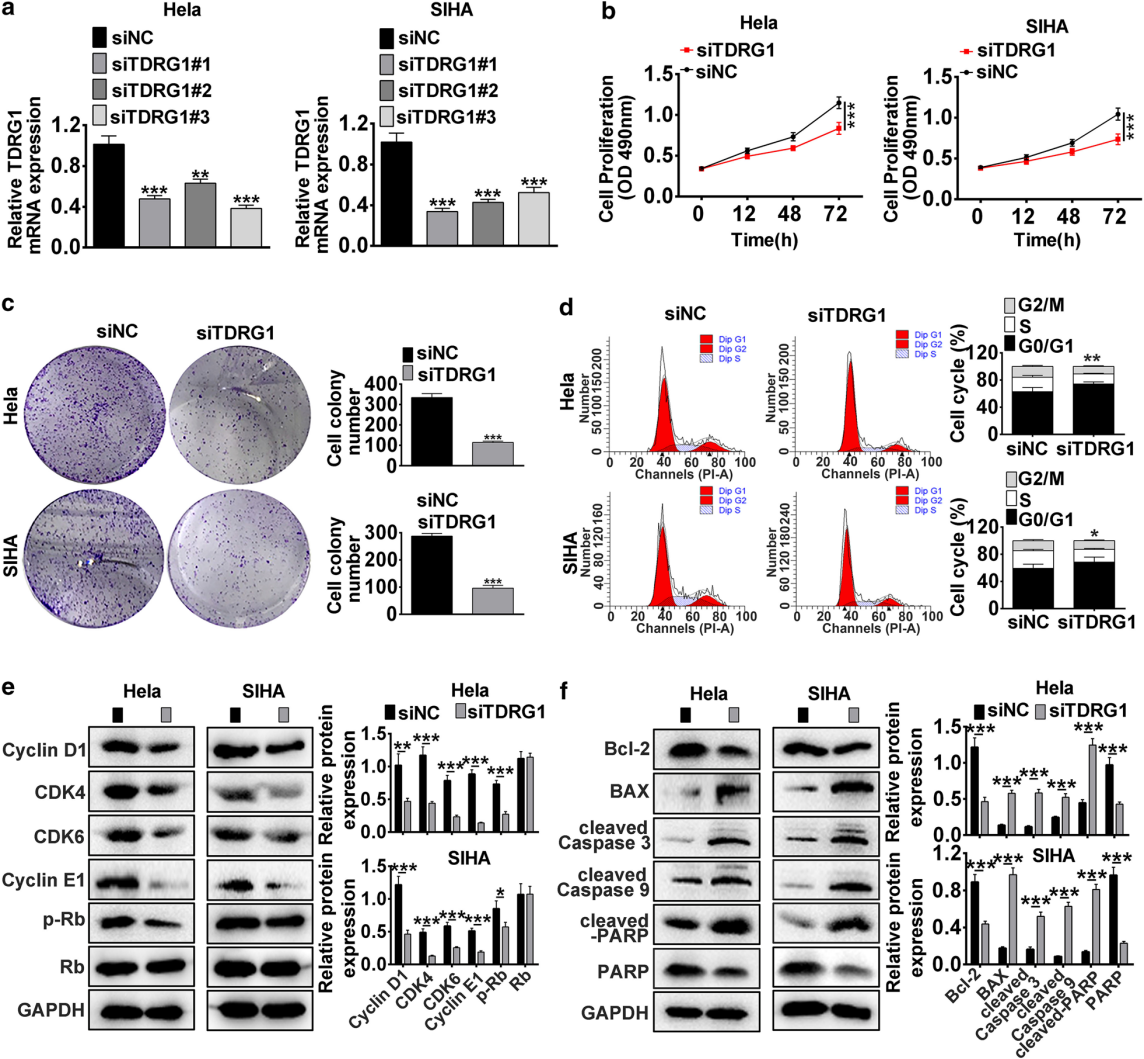
Knockdown of TDRG1 suppressed cell proliferation in Hela and SIHA cells. **a** The expression levels of TDRG1 were examined by qRT-PCR in Hela and SIHA cells transfected with siRNAs targeted TDRG1 (siTDRG1#1, siTDRG1#2, siTDRG1#3). **b** Growth curves were analyzed by MTT assay in Hela and SIHA cells transfection with siTDRG1 and siNC for 0, 12, 48 and 72 h. **c** The effect of TDRG1 silencing on Hela and SIHA cell proliferation were determined by colony formation assay. **d** The effect of down-regulated TDRG1 on the cell cycle of Hela and SIHA cells were analyzed by flow cytometry assay. The cell cycle distribution was exhibited. **e** The influence of siTDRG1 on the protein levels including Cyclin D1, CDK4, CDK6, Cyclin E1, p-Rb and Rb were detected by western blot in Hela and SIHA cells. **f** The effect of silencing of TDRG1 on the apoptotic proteins-Bcl-2, BAX, cleaved Caspase 3, cleaved Caspase 9, cleaved PARP and PARP were checked by western blot in Hela and SIHA cell lines. Data were expressed as mean ± SD. **P < 0.01, ***P < 0.001

Further, loss of function experiments was performed to examine the role of TDRG1 in Hela and SIHA cell lines. Firstly, three siRNAs targeting the CDS region of TDRG1 were transfected into CC cell lines to checkr their knockdown efficiency. As shown in Fig. 2a, siTDRG1#1, siTDRG1#2 and siTDRG1#3 remarkably decreased the expression of TDRG1 in Hela and SIHA cell lines. siTDRG1#1 (siTDRG1) was chosen for the further study as its higher downregulation efficiency (*P* < 0.01, *P* < 0.001). MTT assay demonstrated that interference of TDRG1 decreased the capacity of cell proliferation in Hela and SIHA cell lines (*P* < 0.001, Fig. 2b). Colony formation assay showed that down-regulated expression of TDRG1 inhibited the colonies number of Hela and SIHA cells (Fig. 2c). Flow cytometry analysis of the cell cycle indicated that the proportion of S phase dramatically declined after silencing of TDRG1 expression in Hela and SIHA cell lines (Fig. 2d). In addition, cyclin-dependent kinase 4/6 (CDK4/6) correlation with the D-type cyclins and phosphorylating the retinoblastoma protein (Rb) plays important roles in G_0/1_/S phase transition [26]. We measured the protein levels CDK4, CDK 6, Cyclin D1, Cyclin E1 and Retinoblastoma (pRb) using western blot assay. The western-blot assay results showed that the protein levels of Cyclin D1, CDK4, CDK6, Cyclin E1, p-Rb were notably decreased by TDRG1 siRNA compared with siNC group in Hela and SIHA cells (*P* < 0.05, *P* < 0.01, *P* < 0.001, Fig. 2e), whereas no alteration of Rb expression was detected (*P* < 0.05, Fig. 2e). As shown in Fig. 2f, silencing of TDRG1 increased the expressions of pro-apoptotic proteins-BAX, cleaved Caspase 3, cleaved Caspase 9 and cleaved-PARP, but decreased the expression of PARP and the anti-apoptotic protein-Bcl-2 levels compared with siNC treatment in Hela and SIHA cells (*P* < 0.001). These data suggested that knockdown of TDRG1 inhibited the G1/S phase transition and promoted the apoptosis in CC cell lines.

**Fig. 3:**
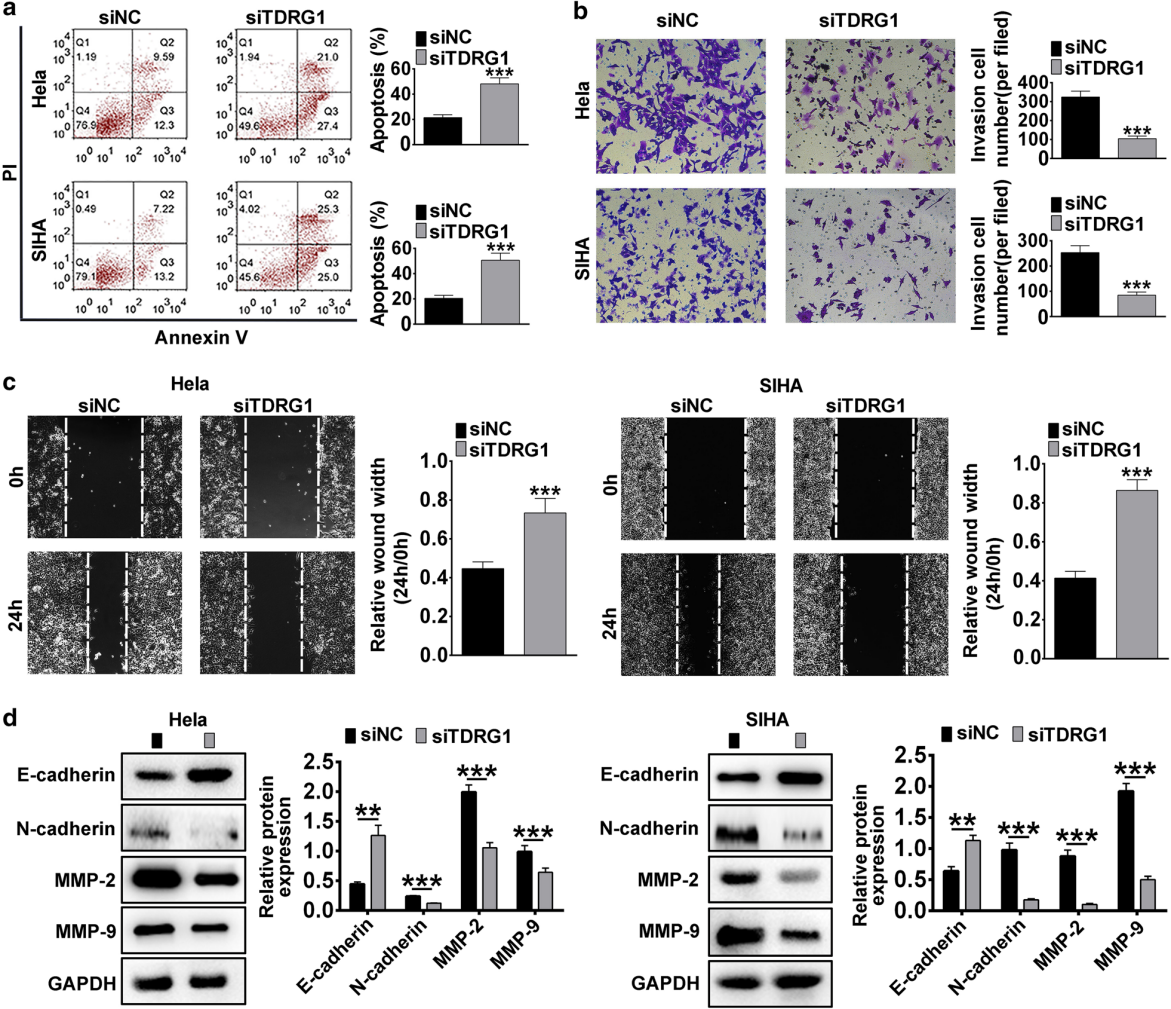
Silencing of TDRG1 inhibited cell migration and invasion in Hela and SIHA cells. **a** The role of knockdown of TDRG1 expression on Hela and SIHA cells the apoptosis was checked by flow cytometry assay. **b** The function of decreased TDRG1 on the migration ability of Hela and SIHA cells were determined by wound-healing assay. **c** The effect of TDRG1 silencing on the invasion ability of Hela and SIHA cells were determined by transwell assay. **d** The influence of siTDRG1 on the expressions of E-cadherin, N-cadherin, MMP-2 and MMP-9 were measured using western blot in Hela and SIHA cells. Data were expressed as mean ± SD. **P < 0.01, ***P < 0.001

Further, the apoptotic cells ratio was markedly elevated in Hela and SIHA cell lines transfected with siTDRG1 compared with siNC (*P* < 0.001, Fig. 3a), indicated by flow cytometry analysis. Moreover, the transwell assay confirmed that the invasion cell numbers of Hela and SIHA cells treated with siTDRG1 were remarkably decreased compared with siNC group (*P* < 0.001, Fig. 3b). The wound-healing assay proved that cells transfected with siTDRG1 exhibited a slower closing of scratch wound compared with siNC group in Hela and SIHA cell lines (*P* < 0.001, Fig. 3c). In addition, we also detected the expressions of E-cadherin, N-cadherin, Matrix metalloproteinase (MMP)-2 along with MMP-9, which are closely associated with tumor metastasis capacity by western-blot [27]. As shown in Fig. 3d, knockdown TDRG1 enhanced the expression of epithelial marker E-cadherin, while reduced mesenchymal marker N-cadherin, invasion related proteins-MMP-2, MMP9 compared with siNC group in CC cells (*P* < 0.01, *P* < 0.001). Taken together, these data suggested that knockdown of TDRG1 inhibited cell proliferation, migration and invasion in CC cell lines.

### TDRG1 is negatively correlated with miR-326

**Fig. 4:**
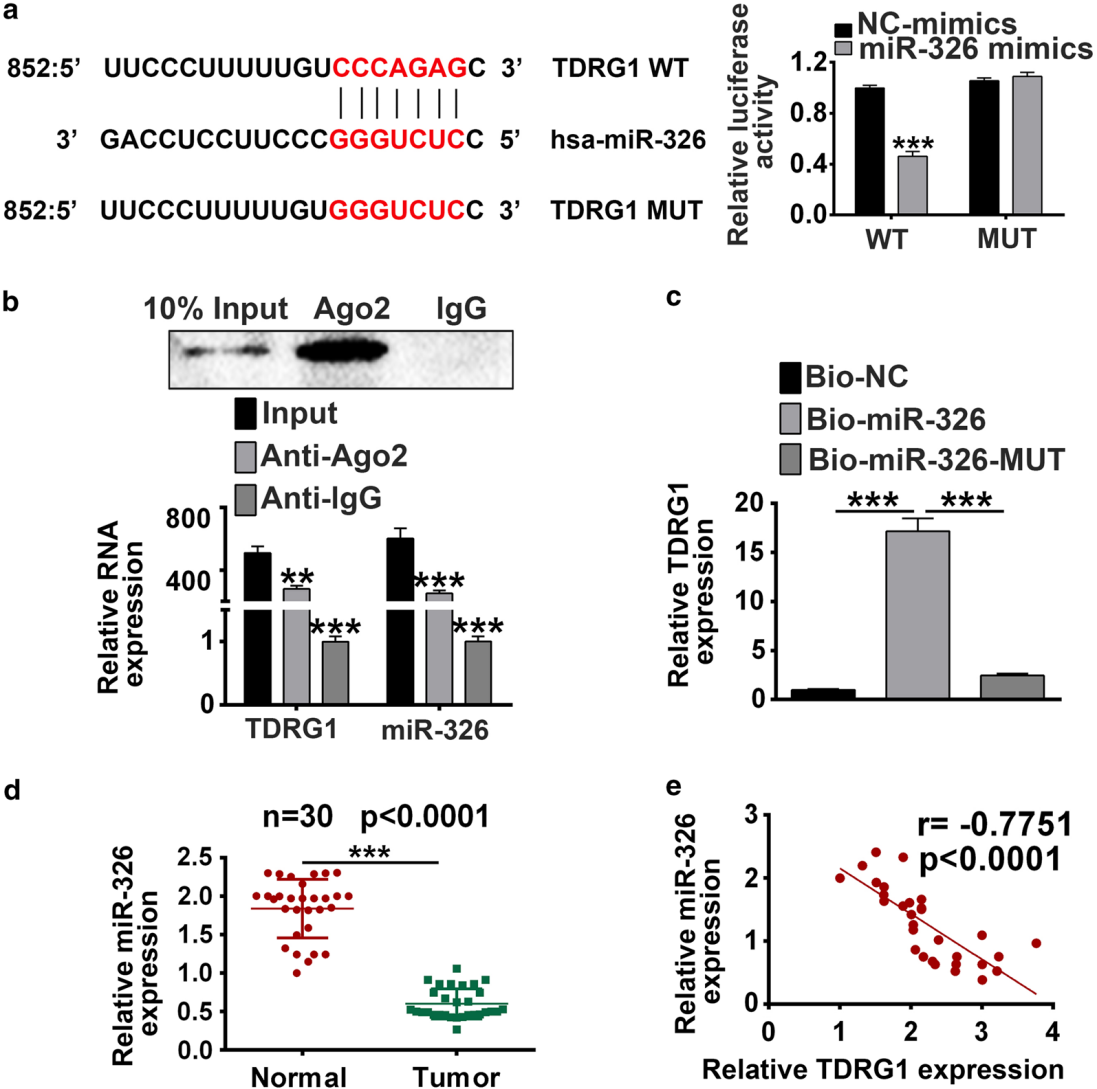
TDRG1 inversely interacted with miR-326. **a** TDRG1 mRNA wide-type (TDRG1 WT) and the mutated-type (TDRG1 MUT) in the miR-326 binding sites were shown. Luciferase activity of HEK293T cells co-transfected with miR-326 mimics or NC mimics and luciferase reporters containing TDRG1 WT or TDRG1 MUT transcript were determined by dual-luciferase reporter assays. **b** Endogenous miR-326 precipitated by AGO2 upon overexpression of TDRG1 was determined by RIP assay in HEK293 cells. **c** The interaction of TDRG1 and miR-326 in HEK293 cells was detected by RNA pull-down assay which precipitated with bio-miR-326, bio-miR-326 Mut or bio-NC, and then the expression of TDRG1was checked by qRT-PCR. **d** The expression of miR-326 in CC tissues and corresponding normal tissues were detected by qRT-PCR. n = 30. **e** The correlation between TDRG1 and miR-326 was analyzed. Data were expressed as mean ± SD. **P < 0.01, ***P < 0.001

Recently, miRNAs are considered to be key modulator involved in the role of lncRNA [13, 14]. To investigate the mechanism of the effect of TDRG1 on CC cells, we screened miRNAs that have complementary base paring with TDRG1 using online software program miRBase (http://www.mirbase.org/). We identified miR-326 as a potential target miRNA for TDRG1 (Fig. 4a), which confirmed by luciferase reporter assays. We generated TDRG1 wild-type (TDRG1 WT) luciferase plasmids containing the potential miR-326 binding sites as well as a mutated version of each site (TDRG1 MUT) (Fig. 4a). The luciferase activity of cells transfected with TDRG1 WT plasmid was significantly decreased by miR-326 mimics (*P* < 0.001), while there was no difference in cells transfected with TDRG1 MUT plasmid (*P* > 0.05, Fig. 4a). This result indicated the targeting relationship between miR-326 and TDRG1. Further, to validate the direct interaction between TDRG1 and miR-326, RIP and RNA pull down assay were performed. The endogenous miR-326 expression was specifically enriched in the cells transfected with TDRG1 overexpression plasmid compared with IgG control (*P* < 0.001, Fig. 4b). Meanwhile, as shown in Fig. 4c, endogenous TDRG1 was efficiently pulled down by bio-miR-326 (*P* < 0.001) but not miR-326 Mut (*P* < 0.001). The expression of miR-326 was decreased in cervical tumor tissues compared with normal tissues (*P* < 0.001, Fig. 4d). In addition, the expression of TDRG1 and miR-326 exhibited a dramatically negative correlation confirmed by Spearman’s correlation analysis (*P* < 0.001, Fig. 4e) in CC tissues. Altogether, these data demonstrated that TDRG1 is targeted direct target of miR-326 and negatively regulated by miR-326.

### TDRG1 positively regulated MAPK1 expression via miR-326

**Fig. 5:**
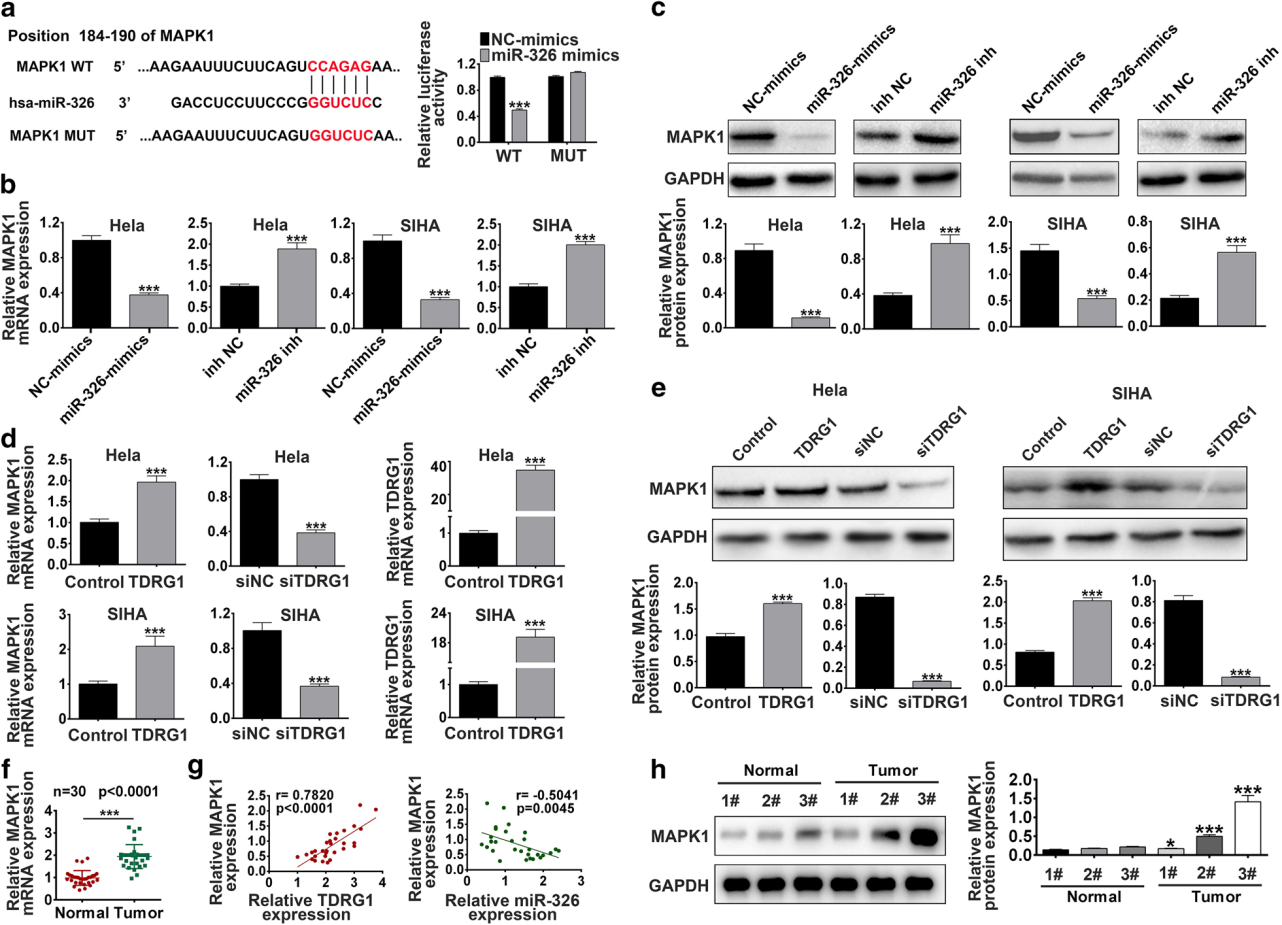
MAPK1 was directly targeted by miR-326 and positively regulated by TDRG1. **a** MAPK1 3′-UTR wide-type (MAPK1 3′-UTR WT) and the mutated-type (MAPK1 3′-UTR MUT) in the miR-326 binding sites were shown. Luciferase activitis of HEK293 cells co-transfected with miR-326 mimics or NC mimics and luciferase reporters containing MAPK1 3′-UTR WT or MAPK1 3′-UTR MUT transcript were determined by dual-luciferase reporter assays. **b**, **c** The levels of MAPK1 in Hela and SIHA cells transfected with miR-326 mimics or NC mimics, as well as miR-326 inhibitor or NC inhibitor were analyzed by qRT-PCR and western blot. **d**, **e** The expression levels of TDRG1 were detected in Hela and SIHA cells treated with TDRG1 overexpression or control plasmid using qRT-PCR assay. The levels of MAPK1 in Hela and SIHA cells transfected with TDRG1 overexpression or control plasmid, as well as siTDRG1 or siNC were analyzed by qRT-PCR and western blot. **f** The expression of MAPK1 in CC tissues and corresponding normal tissues were detected by qRT-PCR. n = 30. **g** The correlation between MAPK1 and TDRG1, as well as MAPK1 and miR-326 expression was analyzed. **h** The protein level of MAPK1 in CC patients and normal subjects were measured by western blot. n = 3. Data were expressed as mean ± SD. *P < 0.05, ***P < 0.001 r

Subsequently, bioinformatics tool Targetscan (http://www.targetscan.org/) predicted that miR-326 shared complementary binding sites with 3′UTR of MAPK1 mRNA (Fig. 5a). We generated MAPK1 3′-UTR wild-type (MAPK1 3′-UTR WT) luciferase plasmids containing the potential miR-326 binding sites as well as a mutated version of each site (MAPK1 3′-UTR MUT) (Fig. 5a). Dual luciferase reporter assay results demonstrated that the luciferase activity of cells transfected with MAPK1 3′UTR WT was significantly decreased by miR-326 mimics (*P* < 0.001), while there was no alteration in MAPK1 3′UTR MUT transfected group (*P* > 0.05, Fig. 5a). Further, qRT-PCR results proved that the mRNA level of MAPK1 in Hela and SIHA cells were significantly decreased by miR-326 mimics (*P* < 0.001), while increased by miR-326 inhibitor (*P* < 0.001, Fig. 5b). Meanwhile, western blot also confirmed that the MAPK1 protein level was reduced or enhanced by miR-326 mimics or inhibitor, respectively in Hela and SIHA cells (*P* < 0.001, Fig. 5c). These data suggested that MAPK1 was a direct target of miR-326. Next, to verify the targeting relationship between TDRG1 and MAPK1, the expression of TDRG1 was confirmed to be up-regulated by TDRG1 plasmid in Hela and SIHA cells (Fig. 5d), further, qRT-PCR and western blot were performed and the results revealed that MAPK1 mRNA level and protein level were up-regulated in Hela and SIHA cells when transfected with MAPL overexpression plasmid TDRG1 (*P* < 0.001), while down-regulated by siTDRG1 (*P* < 0.001, Fig. 5d, e). We also found that MAPK1 mRNA was highly expressed in cervical tumor tissues compared with normal tissues (*P* < 0.001, Fig. 5f). Pearson’s correlation analysis proved that TDRG1 expression was positively while miR-326 expression was inversely correlated with MAPK1 expression in CC tissue samples (TDRG1: *P* < 0.001; miR-326: *P* < 0.01, Fig. 5g). Further, the protein level of MAPK1 was up-regulated in cervical tumor tissues compared with normal tissues (*P* < 0.05, *P* < 0.001, Fig. 5h). Therefore, these results proved that MAPK1 expression is inhibited by miR-326, while positively modulated by TDRG1.

### TDRG1 promoted cell proliferation, migration and invasion via targeting miR-326

**Fig. 6:**
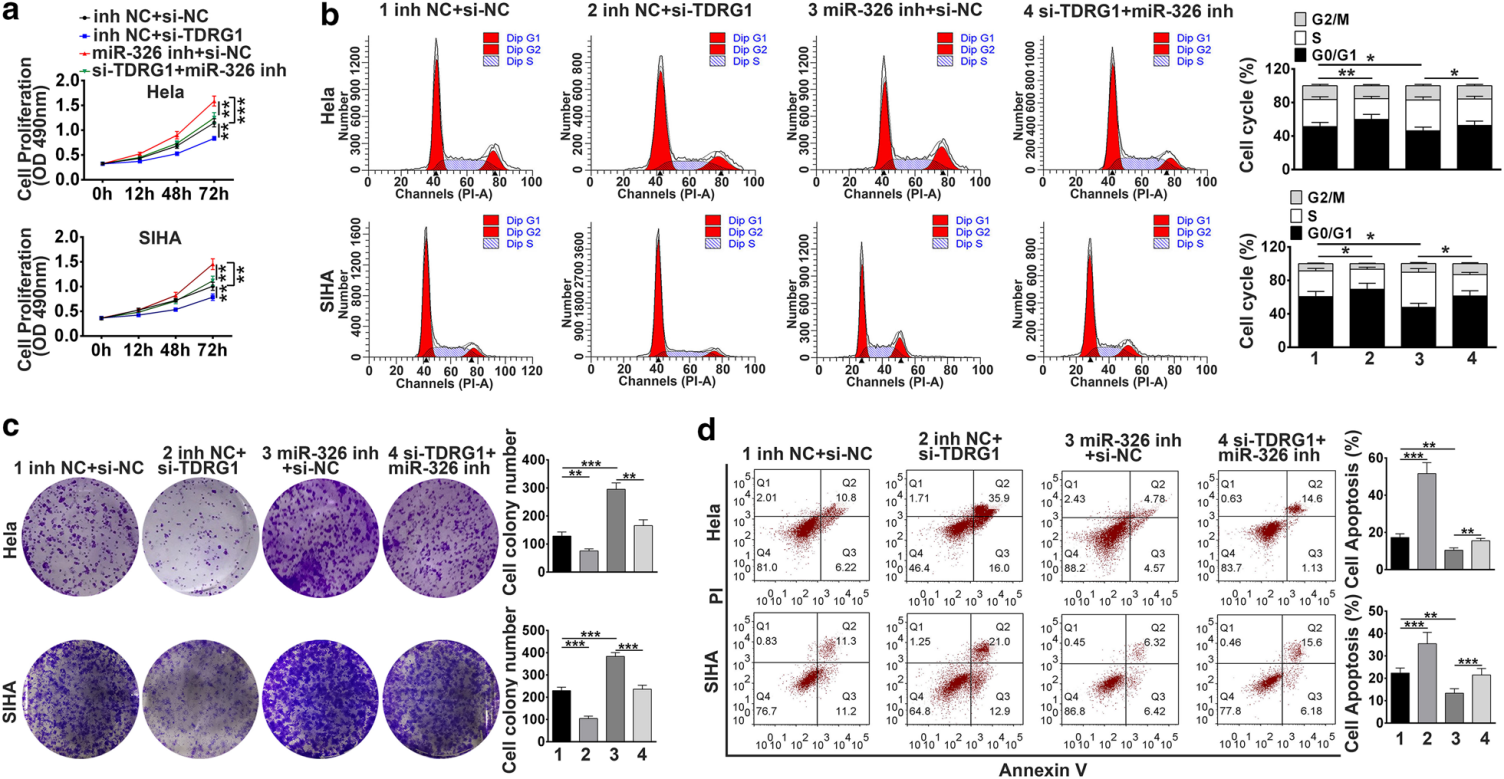
TDRG1 modulated cell proliferation, migration and invasion in Hela and SIHA cell lines via miR-326. **a**, **c** The cell proliferation abilities were determined by MTT assay, and colony formation assays. **b**, **d** Cell cycle and cell apoptosis of Hela and SIHA cell lines co-transfected with siTDRG1 or siNC and miR-326 inhibitor or inhibitor NC were analyzed by flow cytometry assay. Data were expressed as mean ± SD. **P < 0.01, ***P < 0.001

**Fig. 7:**
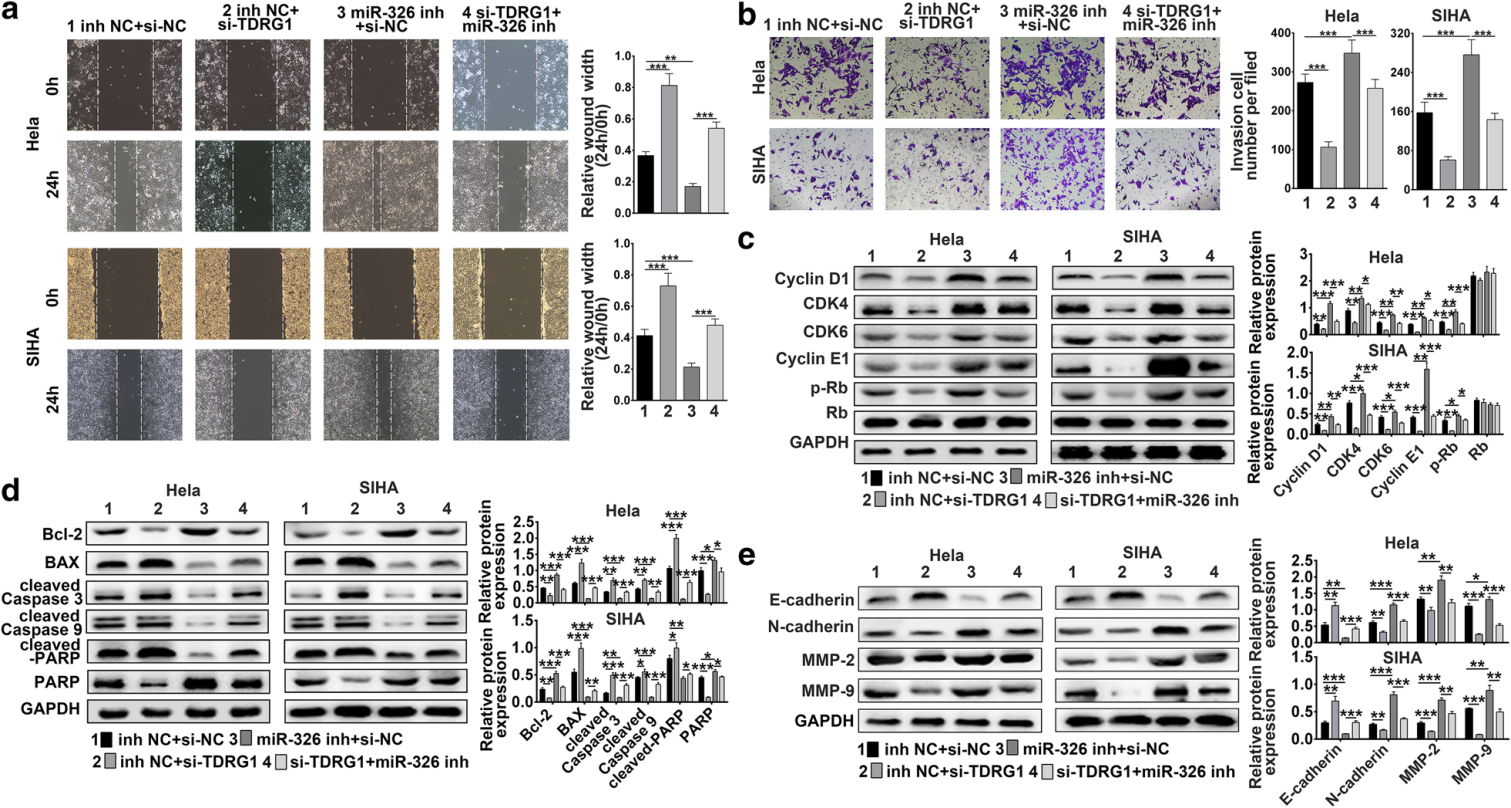
TDRG1 regulated cell migration and invasion in Hela and SIHA cell lines by targeting miR-326. **a**, **b** The migration and invasion abilities were measured by wound-healing assay and transwell migration assay in Hela and SIHA cell lines, which were co-transfected with siTDRG1 or siNC and miR-326 inhibitor or NC inhibitor. **c**–**e** The cell cycle related proteins (**c**), anti- or pro- apoptotic factors (**d**) and metastasis related proteins (**e**) were detected using western blot in CC cell lines co-treated with siTDRG1 or siNC and miR-326 inhibitor or NC inhibitor. Data were expressed as mean ± SD. *P < 0.05, **P < 0.01, ***P < 0.001

To explore whether TDRG1 exerted its function via miR-326, Hela and SIHA cells were co-transfected with TDRG1 siRNA and miR-326 inhibitor. MTT and colony formation assay results showed that the inhibitory effect of cell proliferation ability by interference of TDRG1 was abrogated by miR-326 inhibitor (*P* < 0.01, *P* < 0.001, Fig. 6a, c). Then flow cytometry analysis was performed to detect the effect of downregulation of TDRG1 and miR-326 on cell cycle and cell apoptosis. The results suggested that miR-326 knockdown completely reversed the down-regulation of TDRG1-induced decrease of the cell population in S phase and decrease of cell apoptosis (*P* < 0.01, *P* < 0.001, Fig. 6b, d). More convincingly, in CC cells, the wound-healing and transwell assay were carried out and the results proved that cells transfected with siTDRG1 inhibited migration and invasion abilities, while this effect was attenuated by miR-326 inhibitor (*P* < 0.01, *P* < 0.001, Fig. 7a, b). In addition, the siTDRG1 transfection-induced inhibition protein level of cell cycle related proteins-Cyclin D1, CDK4, CDK6, Cyclin E1, p-Rb induced by were reversed by miR-326 inhibitor treatment (*P* < 0.05, *P* < 0.01, *P* < 0.001, Fig. 7c). The expression of pro-apoptotic proteins were increased by siTDRG1 but decreased by miR-326 inhibitor (*P* < 0.05, *P* < 0.01, *P* < 0.001), while the anti-apoptotic protein-Bcl-2 level presented contrary trends (*P* < 0.01, *P* < 0.001, Fig. 7d). The enhanced level of E-cadherin treated by siTDRG1 was attenuated by miR-326 inhibitor (*P* < 0.01, *P* < 0.001), meanwhile, silencing of TDRG1 induced lower expressions of N-cadherin, MMP-2 and MMP-9 were further elevated by miR-326 inhibitor (*P* < 0.05, *P* < 0.01, *P* < 0.001, Fig. 7e). Thus, these results indicated that miR-326 was essential for the inhibitory effect of down-regulated TDRG1 on CC cell growth, migration and invasion.

### Knockdown of TDRG1 suppressed CC cells tumorigenesis via enhancing miR-326 and decreasing MAPK1 level in vivo

**Fig. 8:**
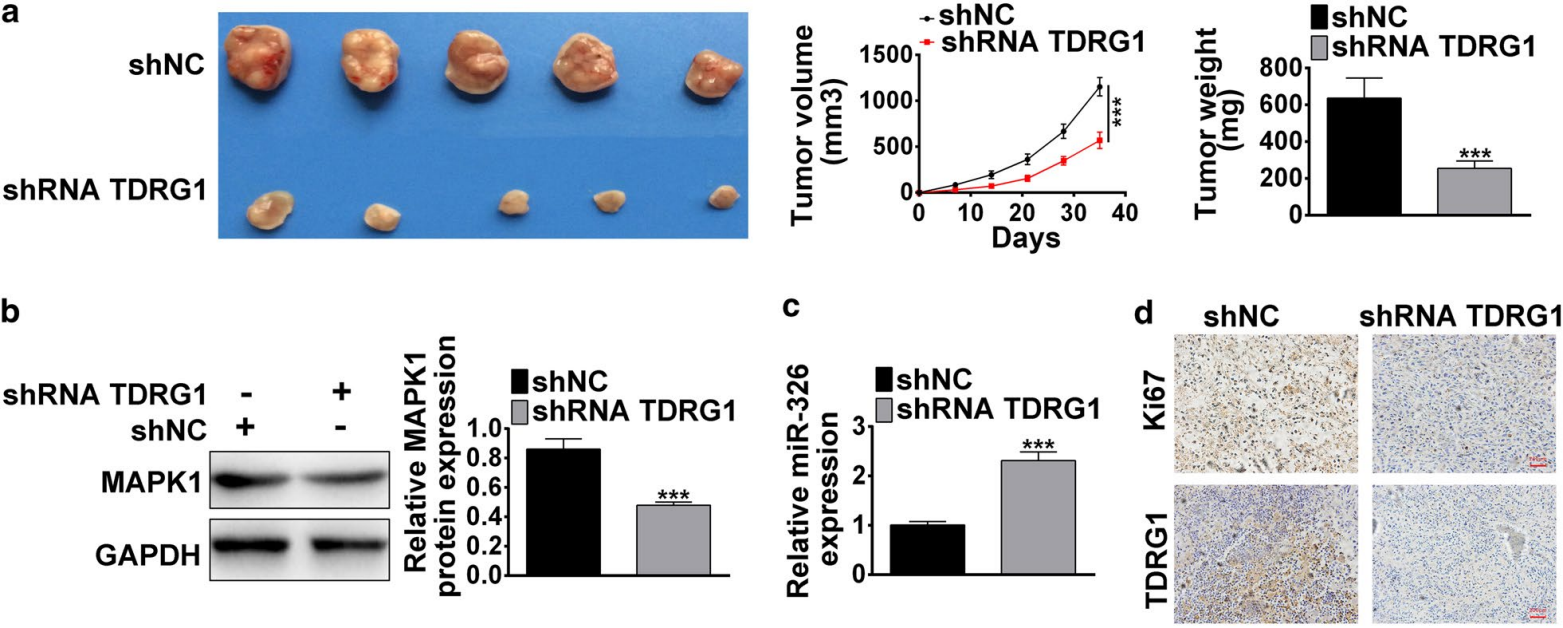
Down-regulated of TDRG1 expression inhibited CC cell growth through reducing MAPK1 level in vivo. Mice were inoculated with Hela cell line stably transfected with shRNA TDRG1 or shNC plasmid. **a** Tumors collected from mice were shown. The effect of TDRG1 silencing on tumor volume curve and tumor weight was analyzed. **b** The effect of decreased TDRG1 on the MAPK1 protein level in vivo was measured by western blot. **c** The role of interference of TDRG1 on the miR-326 expression level in vivo was measured by qRT-PCR. **d** The proliferation marker Ki67 and TDRG1 expression level in vivo were detected by IHC assay. Representative photos were shown. Bar = 200 μm. Data were expressed as mean ± SD. ***P < 0.001

Further, orthotopic xenograft mouse models were introduced to assess the effect of TDRG1 on CC cell tumorigenesis in vivo. Hela cells stably transfected with shRNA TDRG1 or shNC plasmid were subcutaneously inoculated into male nude mice. As shown in Fig. 8a, the tumor growth and tumor size in shRNA TDRG1 group were markedly slower and smaller than shNC group (*P* < 0.001). After 5 weeks, the tumor weight was dramatically reduced in shRNA TDRG1 treated group compared with shNC group (*P* < 0.001, Fig. 8a). Western blot and qRT-PCR were performed to check the miR-326 and MAPK1 level in vivo. Compared with siNC group, the protein level of MAPK1 was decreased (*P* < 0.001, Fig. 8b), while miR-326 level was increased in shRNA TDRG1 group (*P* < 0.001, Fig. 8c). Further, the expression of TDRG1 and proliferative indicator Ki67 were detected by IHC assay. The results suggested that, in xenograft tumors, the expression level of Ki67 and TDRG1 were significantly lower in shRNA TDRG1 group compared with siNC group (Fig. 8d). Collectively, these data indicate that knockdown of TDRG1 inhibited CC cell growth and tumorigenesis in vivo.

## Discussion

CC is the one of the most frequent gynaecological malignancies in females and causes notably morbidity and mortality worldwide [28, 29]. Because of the delayed diagnosis and poor therapeutic treatment, it is urgently screened available biomarkers for clinically diagnosis and treatment of CC patients. Recently, a variety of lncRNAs are proved to be abnormally expressed in CC and contribute to the pathological progress of CC via acting as oncogenes or tumor suppressors [30, 31]. In present study, we firstly found that lncRNA TDRG1 was highly expressed in CC patients’ tissues and CC cell lines compared with normal tissue and non-cancer cells. Interference of TDRG1 by its siRNA suppressed cell proliferation, migration and invasion in CC cell lines. TDRG1 was proved to be interacted with miR-326 and positively regulated MAPK1, which is a target of miR-326. Finally, we demonstrated that TDRG1 participated in regulation of CC progression via regulation of miR-326/MAPK1 axis and could be a potential biomarker in CC prognosis.

TDRG1 was firstly identified as a testicular-specific gene and encodes a 100 amino acid protein without any characterized protein domain [8]. It exclusively expressed in the testis in non-reproductive tissues, and plays a major role in regulating human spermatogenesis and sperm motility [8, 9]. With the in-depth research, TDRG1 was considered as a key regulator in reproductive organ related cancer such as testicular germ cell tumors (TGCT) [10, 32, 33], epithelial ovarian carcinoma [11] and endometrial carcinoma [12]. Down-regulated expression of TDRG1 reduced the biological activity of TGCT cells [10], and enhanced proliferation and migration of seminoma cells through modulation of the PI3 K/Akt/mTOR signaling pathway and mitochondria-mediated apoptotic pathway [32, 33]. Furthermore, as a lncRNA, TDRG1 enhanced tumorigenicity by inhibiting the miR-93/RhoC pathway in epithelial ovarian carcinoma [11] and promoted endometrial carcinoma cell development and invasion by positively targeting VEGF-A in endometrial carcinoma [12]. However, there is no evidence that TDRG1 involved in regulation of the pathological process in CC. The present study demonstrated that TDRG1 was up-regulated in CC tissue and was related with poor clinical outcome of CC patients. Moreover, TDRG1 was able to enhance the CC cells growth, migration and invasion. Similarly, the expression of TDRG1 was highly expressed in cancerous tissues and acted as an oncogene to activate tumor cells proliferation and invasion in above productive organ related cancer [11, 12, 32]. On the basis, TRDG1 acts as an oncogene and promoter in CC and can be considered as a novel potential therapeutic target. In addition to loss-of-function analysis, gain-of-function studies for overexpressing the TDRG1 level in CC cells is needed to be further explored.

Recently, lncRNAs are widely proved to act as the “sponge” or “ceRNA” in the regulatory network refering to lncRNA, miRNA and target genes [34]. In the current study, we found a metastasis related regulating network which is comprised of TDRG1, miR-326 and MAPK1. In addition, we verified that both TDRG1 and MAPK1 were the targets of miR-326. We demonstrated that TDRG1 could positively modulate MAPK1 expression and mediate invasiveness by working as a ceRNA of miR-326. Previous studies reported that miR-326 works as a tumor suppressor [35, 36] and also acts as a ceRNA or sponge RNA of lncRNA in various cancers [20, 37]. Specifically, miR-326 is involved in regulation of CC progression. LncRNA-HOTAIR promotes cell growth and metastasis by inhibition miR-326 expression [20], whereas up-regulated miR-326 suppressed CC cell tumor tumorigenicity via targeting Elk-1 [19]. Furthermore, VEGF-C promoted CC invasion by attenuating miR-326 expression and enhancing cortactin level via c-Src signaling [21]. Consistent with these studies, our study showed that TDRG1 and miR-326 are negatively modulated each other by directly binding, and the downregulation of TDRG1 expression inhibited cell proliferation and metastasis via eliminating restrain of miR-326 expression in CC. In other words, miR-326 acts as a tumor suppressor in TDRG1 regulation of tumorigenicity in CC. It is maybe interesting to demonstrate the effect of co-enhance the expression TDRG1 and miR-326 in CC in the future.

Numerous studies have shown that MAPK1 plays pivotal roles in a variety of cancers especially CC. It is considered as an oncogene targeted by miRNA or regulated by lncRNA to enhance CC progression [38–40]. For instance, miR-329-3p exhibited a critical tumor suppression role by directly suppressing MAPK1 in CC [39]. LncRNA HOTAIR activated cell growth and metastasis in CC via targeting miR-23b/MAPK1 axis [40]. However, there was no evidence suggested that MAPK1 is a target of TDRG1 or miR-326. We currently reported that MAPK1 was negatively correlated with miR-326 but positively regulated by TDRG1. Further studies confirmed that the TDRG1 activated CC growth and metastasis by inhibition of miR-326 and increasing of MAPK1 in vivo. Consistent with above studies, MAPK is also considered as an oncogene in CC pathological development.

## Conclusion

In conclusion, we firstly demonstrated lncRNA TDRG1 was up-regulated in CC, and TDRG1 played important role in promoting CC aggressiveness. Mechanically, down-regulation of TDRG1 enhanced the expression level of miR-326 and in turn decreased MAPK1 levels, thereby alleviating CC cell proliferation and invasion. TDRG1 exerts its oncogenic characteristic and our studies provides a novel molecular basis for potential applications of TDRG1 in the prognosis and treatment of CC.

## Data Availability

Not applicable.

## Abbreviations

CC: cervical cancer
lncRNAs: long noncoding RNAs
TDRG1: testis developmental related gene 1
ceRNAs: competing endogenous RNAs
UTR: untranslated region
